# Molecular Design and Mechanism Analysis of Phthalic Acid Ester Substitutes: Improved Biodegradability in Processes of Sewage Treatment and Soil Remediation

**DOI:** 10.3390/toxics10120783

**Published:** 2022-12-13

**Authors:** Shuhai Sun, Qilin Zuo, Meijin Du, Yu Li

**Affiliations:** 1 School of Hydraulic and Environmental Engineering, Changchun Institute of Technology, Changchun 130012, China; 2 MOE Key Laboratory of Resources and Environmental Systems Optimization, North China Electric Power University, Beijing 102206, China

**Keywords:** phthalic acid esters, 3D-quantitative structure-activity relationship, microbial degradation of sewage and soil, photodegradation, endocrine disrupting toxicity, molecular docking

## Abstract

Phthalic acid esters (PAEs) have the characteristics of environmental persistence. Therefore, improving the biodegradability of PAEs is the key to reducing the extent of ecological harm realized. Firstly, the scoring function values of PAEs docking with various degrading enzymes in sewage treatment were calculated. Based on this, a 3D-quantitative structure-activity relationship (3D-QSAR) model for PAE biodegradability was built, and 38 PAE substitutes were created. By predicting the endocrine-disrupting toxicity and functions of PAE substitutes, two types of PAE substitutes that are easily degraded by microorganisms, have low toxicity, and remain functional were successfully screened. Meanwhile, the differences in the mechanism of molecular degradation difference before and after PAE modification were analyzed based on the distribution characteristics of amino acid residues in the molecular docking complex. Finally, the photodegradability and microbial degradability of the PAE substitutes in the soil environment was evaluated. From the 3D-QSAR model design perspective, the modification mechanism of PAE substitutes suitable for sewage treatment and soil environment degradation was analyzed. We aim to improve the biodegradability of PAEs at the source and provide theoretical support for alleviating the environmental hazards of using PAEs.

## 1. Introduction

Phthalate esters (PAEs) are a kind of substitutes synthesized by esterification of phthalic anhydride with various alcohol compounds, which have high fat solubility and low water solubility [1,2]. PAEs can be detected in water, soil, and air environmental media as these are widely used and produced in bulk [1]. In addition, the toxic effect of ecological hormones, the toxicity of reproductive development, carcinogenesis, teratogenesis, and mutagenicity of PAEs have attracted wide attention worldwide [3]. According to earlier research, it takes approximately 400 years for plastics to degrade completely in water and soil. PAEs, the primary additives in plastics, are continuously released into the environment during plastic degradation. PAE degradation is primarily dependent on microbiological and photodegradation processes, but the degradation rate is slow, and the degree of mineralization is low [4]. It is difficult to degrade PAEs in the absence of ultraviolet light in water sediments [5]. The refractory nature of PAEs results in ecological hazards [6]. Therefore, improving the environmental degradability of PAEs while retaining their functional integrity is of great importance.

Although PAEs have low water solubility, a small part of them are still soluble in water. Due to atmospheric deposition, soil leaching, and domestic drainage, PAEs are widely accumulated in water bodies, and high levels of PAEs have been detected in sewage [7]. The sewage treatment process serves as an essential transit point for the discharge of PAEs into the environment, and physical, chemical, and microbiological degradation methods are three major ways of treating sewage [6]. PAEs are stable in the environment. The physicochemical properties of PAEs vary greatly, and their biodegradability is low. Therefore, it is difficult to remove PAEs effectively [8]. At low concentrations, conventional physical or chemical methods are adequate for removing PAEs. Secondary contamination is possible, and the efficiency of the methods is greatly influenced by external environmental conditions such as temperature and pH [9]. It has been found that more than 80 degrading bacteria can degrade PAEs, and 50% of these bacteria can degrade multiple PAEs simultaneously [10]. The main PAE pollutants in the environment, dibutyl phthalate (DBP) and bis(2-ethylhexyl) phthalate (DEHP), are typically degraded by mixed strains. PAEs are typically degraded by the synergistic action of mixed strains, but in the absence of bioenhancers or without the use of intelligent control methods, these cannot be quickly removed. Aerobic and anaerobic biodegradation methods are the two types of microbial degradation methods currently used for wastewater treatment [11]. Aerobic biodegradation is primarily carried out by nitrite bacteria, Escherichia coli, yeast, and Pseudomonas adaceae, and anaerobic biodegradation is primarily carried out by denitrifying bacteria, denitrifying bacilli, hydrolytic acidifying bacteria, and acetogen [11]. During aerobic biodegradation, mixed aerobic microorganisms exert a degrading effect on PAEs. The degradation rate decreases with an increase in the molecular chain, and the degree of mineralization is low [12]. During anaerobic biodegradation, anaerobic microorganisms primarily use PAEs as a carbon source for growth and reproduction, thereby utilizing PAEs for degradation [12]. Aquatic conditions have a strong influence on the anaerobic biodegradation process. PAEs that cannot be removed effectively during the sewage treatment enter the surface water environment via secondary biochemical effluent. This has a knock-on effect on the aquatic ecosystem and human health [13]. The disadvantages of the sewage treatment process are the long construction period, high investment cost, and low process level [14]. It is difficult to improve the removal rate of PAEs by improving the sewage treatment process. Therefore, improving the microbial degradability of PAEs is one of the keys to realizing the effective degradation of PAEs during sewage treatment.

A soil environment is important for PAE migration and storage [15]. DEHP and DBP are the primary pollutants in soil [16], and these are produced during reclaimed water irrigation [13], atmospheric dry and wet deposition [17], and leaching of accumulated waste plastics and agricultural plastic film residues [16,18]. The distribution of PAEs in the soil is affected by physical and chemical properties, environmental conditions, and tillage methods [19]. Long-chain DEHP molecules are characterized by high molecular weights, high octanol-water partition coefficients, and low water solubility. These can be readily adsorbed by soil, causing harm to soil ecology [19]. PAE degradation in the soil environment is primarily caused by photodegradation and microbiological degradation. Among them, microbial degradation is distinguished by its high biosafety. Nonetheless, soil temperature, physical properties, and chemical properties can easily limit the degradation process [20]. The photodegradation of PAEs is primarily achieved in the presence of solar energy and various photosensitive substances in the soil. The substances are degraded directly through the absorption of UV light [21]. However, the UV content in sunlight is low, the photoreduction rate is slow, and the photosensitive substances are prone to failure during degradation [22,23]. Therefore, PAEs could not be effectively removed following photodegradation and microbial degradation methods. Residual PAEs in the soil environment can enter the ecosystem directly or indirectly and migrate through the food chain to the human body, posing a potential threat to human health [15]. Therefore, improving the degradability of PAEs in sewage and soil environment is of great significance for alleviating the environmental and human health risks posed by PAEs.

We designed and screened easily degradable molecular substitutes for PAEs. We studied: (1) The comprehensive effect value for PAE biodegradability was calculated following the process of molecular docking and the intercriteria correlation (CRITIC) (based on the results from interlayer correlation). A 3D-QSAR model was used to analyze the comprehensive biodegradability of PAEs during water treatment (W-3D-QSAR) and design substitute molecules. (2) The less toxic and easily biodegradable substitutes that exhibit good activity were screened by analyzing the score function value obtained when the PAE substitute molecules and biodegradable complex protein were docked. The QSAR model and the density functional theory (DFT) technique were used to determine the disruptive endocrine toxicity of PAEs (T-QSAR). The molecular docking technique was used to investigate the mechanisms of molecular degradation (before and after PAE modification). (3) The photodegradation and microbial degradation paths of the PAE substitutes were deduced. The energy barrier of the reaction was calculated, and the endocrine disrupting toxicity of the degradation products was predicted to evaluate the photo and biodegradability of the PAE substitutes in soil. (4) To investigate the molecular modification mechanism of PAE substitutes suitable for sewage treatment and soil degradation, the distribution characteristics of the three-dimensional isopotential map of the W-3D-QSAR were determined. The PAEs molecular soil micro-biological degradation 3D-QSAR model (S-3D-QSAR) and T-QSAR were identified. The aim of this study is to design PAE substitutes with high degradability in both sewage treatment and soil, to improve the biodegradability of PAEs themselves from the source, and to provide theoretical guidance for the preparation of environmentally friendly PAEs substitutes in the future.

## 2. Materials and Methods

### 2.1. Screening and Data Source of Molecular Biodegradable Enzymes for PAE Degradation during Sewage and Soil Treatment Processes

Sewage treatment processes and microorganisms in soil primarily rely on the efficiency of the secreted degradation enzymes to effectively degrade PAEs. During the process of sewage treatment, PAEs are primarily degraded by aerobic and anaerobic bacteria. Catalase (PDB ID: 1P80) [24], nitrate reductase (PDB ID: 1HZV), and esterase (PDB ID: 7CUZ), secreted by aerobic bacteria were selected as the primary degrading enzymes for aerobic degradation [25,26], while hydrolase (PDB ID: 2IOF) [27], oxidoreductase (PDB ID: 1NIC), and alcohol dehydrogenase (PDB ID: 3FMX), secreted by anaerobic bacteria, were selected as the primary degrading enzymes for anaerobic degradation [28,29]. Polyphenol peroxidase (PDB ID: 6TOY) [30], hydrolase (PDB ID: 1VA4) [27], hydrolase (PDB ID: 4PSD), and laccase (PDB ID: 6SOO) [30], which were primarily associated with PAE degradation, were chosen to study the degradation process in soil [20,31]. Du et al. identified a composite degradation enzyme (TVSP) consisting of four degradation enzymes using the protein–protein docking technology to comprehensively evaluate the degradation effect of the four degradation enzymes in soil [32]. At the same time, the soil microbial degradation process of the PAE substitutes was evaluated by analyzing the degree of change in the docking scoring function value before and after PAE modification. The relevant data were obtained from the PDB protein structure database (https://www.rcsb.org, accessed on 25 June 2022) of the Brookhaven National Laboratory (Figure S3).

### 2.2. Characterization of the Binding Ability of the PAE Molecules to Microbial Degrading Enzymes Used in the Sewage Treatment Process and Present in the Soil Environment—The Molecular Docking Method

The molecular docking method is widely used to study the interaction between molecules and receptor proteins. It can be used to consider the binding effect of ligands and receptors as a whole [32]. As research objects, we chose 28 PAE molecules (Table 1), and their molecular structures were obtained from the PubMed database (Figure S2). We used the Define and Edit Binding Site module Receptor-Ligand Interactions of Discovery Studio 4.0 software. In the sewage treatment process, twenty-eight PAE molecules were used as ligand molecules to dock with six microbial degrading enzymes (1P80, 1HZV, 7CUZ, 2IOF, 1NIC, and 3FMX). Microbial complex degrading enzymes (TVSP) were selected to study the degradation process in the soil environment [33]. Before conducting the molecular docking process, all degrading enzymes (or the degrading enzyme complexes) were pretreated to remove the ligands, metal ions, and water molecules. Polar hydrogen and point charges were added. At the same time, the Define module was used to find and define the active sites that may bind to the systems. The active site coordinates for 1P80, 1HZV, 7CUZ, 2IOF, 1NIC, 3FMX, and TVSP are (x: 29.042, y: −9.189, z: 89.617), (x: 11.548, y: 16.722, z: 56.172), (x: −29.266, y: −8.789, z: −1.796), (x: −3.683, y: 0.374, z: 25.188), (x: 19.52, y: −1.378, z: −4.238), (x: −81.997, y: 23.841, z: 13.334), and (x: 61.966, y: 29.591, z: 1.939). The dimensions of the docking boxes are all circles with a radius of 15 Å. The docking site of the protein is shown in Figure S1. The PAE molecules were incorporated into the formed active cavity to rapidly dock with the degrading enzyme protein. Finally, the LibDock score was used to analyze the interactions between the ligands and receptors. The higher the docking score function value between the PAE molecules and the degrading enzymes (or composite degrading enzymes), the stronger the degradation ability of the enzymes or the composite degrading enzymes (secreted by microorganisms) [34].

### 2.3. Calculation of Comprehensive Effect Value of Microbial Degradability—The CRITIC Method

As an objective weighting method, the CRITIC method calculates the standard deviation and correlation coefficient between data, assigns weights to different data, and adds and calculates the total effect value of all data using the product of weight and each index [35]. The degradation activity data in this paper were chosen from the docking score function values of 28 PAEs molecules and six degrading enzymes secreted by microorganisms during the sewage treatment process. First, Formula (1), corresponding to the CRITIC method, was used to calculate the standard deviation between the PAE molecules and scoring function values corresponding to different degrading enzymes associated with sewage treatment. Secondly, Formula (2), corresponding to the CRITIC method, is used to calculate the conflict coefficient between other degrading enzymes associated with the sewage treatment process. Using the formula, the critical information on other degrading enzymes is calculated using the estimated standard deviation and conflict coefficient (3). Finally, the weights of the six degrading enzymes used to degrade PAEs were calculated using Formula (4), and the comprehensive effect values of the molecular ability of the six degrading enzymes were calculated using Formula (5). The specific calculation formula for the CRITIC method is as follows:

The standard deviation ($S_{j}$) between the PAE molecules and the scoring function values of the different degrading enzymes associated with the sewage treatment process is calculated as follows:

(1) $$S_{j}=\sqrt{\frac{\sum_{i=1}^{n}{\left(x_{ij}-{\bar{x}}_{j}\right)}^{2}}{n-1}}$$

The correlation coefficient between the different degrading enzymes and the molecular scoring function value corresponding to the PAEs associated with the sewage treatment process ($R_{j}$) is calculated as follows:

(2) $$R_{j}=\sum_{i=1}^{p}\left(1-r_{ij}\right)$$

The vital information on the amount of different microbial degrading enzymes in the sewage treatment process ($C_{j}$) is calculated as follows:

(3) $$C_{j}=S_{j}\sum_{i=1}^{p}\left(1-r_{ij}\right)=S_{j}\times R_{j}$$

The weight of the six degrading enzymes ($W_{j}$) is calculated as follows:

(4) $$W_{j}=\frac{C_{j}}{\sum_{j=1}^{p}C_{j}}$$

The comprehensive effect value corresponding to the simultaneous degradation of the PAE molecules degraded by the six degrading enzymes (Q) is calculated as follows:

(5) $$Q=\sum_{j=1}^{n}W_{j}D_{j},$$

where $x_{ij}$ represents the value of the docking scoring function of the *i* PAE molecules and the j degradation enzyme, ${\bar{x}}_{j}$ represents the average value of the docking scoring function of the 28th PAE molecule for the j degradation enzyme, and $D_{j}$ represents the value of the docking scoring function between the j degradation enzyme and 28 PAE molecules.

### 2.4. Construction of the 3D-QSAR Model for Comprehensive Effects of Biodegradation in PAEs Molecular Sewage Treatment Process—The SYBYL-X Software Method

SYBYL-X 2.0 was used to construct a 3D-QSAR model to study the comprehensive effects of biodegradation. Firstly, we drew the molecular structures of 28 PAEs using the Sketch Molecular module in SYBYL-X 2.0 software. In the Minimize module, we selected the Powell conjugate gradient method and the Tripos molecular force field (max iterations: 10,000; min energy change: 0.005 kcal/mol) and loaded the Gasteiger–Huckel charge. The lowest energy conformations of the PAE molecules were obtained [36]. The training set: test set ratio was set at 3:1 [15], and from the 28 PAE molecules, 21 PAE molecules were randomly selected as the training set, 7 PAE molecules were chosen as the test set, and diisopentyl phthalate (DIPP) molecules were used as the template molecules. The skeleton stacking of 28 PAE molecules was achieved using Align Database in the Applications module. Finally, the combined effect values of the PAE molecules and microbial degrading enzymes were input into the Training table. The W-3D-QSAR model of the PAE molecules was created using the comparative molecular field analysis (CoMFA) method. The types of molecular fields in the CoMFA field are electrostatic field (E) and solid field (S). The leave-one-out method was used to cross-validate the training set compounds, and the cross-validation coefficient q2 and the optimal principal component number N were calculated. Regression analysis was performed through No Validation, and the non-cross validation coefficient r2, standard deviation SEE, and test value F were calculated. Finally, r2pred was calculated from the cross-validation of the test sets. W-3D-QSAR was completed when the above parameters met the modeling requirements [37,38]. The interface scoring function value of the PAE molecules was used to build the S-3D-QSAR, and the microbial complex degrading enzyme (TVSP) in the soil environment was the dependent variable.

## 3. Results and Discussion

### 3.1. Construction and Evaluation of a 3D-QSAR Model to Study the Comprehensive Effects of Microbial Degradation Associated with PAE Removal during Water Treatment

#### 3.1.1. Calculation and Model Construction to Study the Comprehensive Effect Value Associated with Microbial Degradability for the PAE Systems Associated with the Sewage Treatment Process

The scoring function values of 28 PAE molecules and six microbial degrading enzymes (1P80, 1HZV, 7CUZ, 2IOF, 1NIC, and 3FMX) associated with the sewage treatment process were calculated using Discovery Studio 4.0. The CRITIC weight method was used to calculate the comprehensive effect value (Score), as shown in Table 1. W- 3D-QSAR was built using SYBYL-X and the combined effect values of the PAEs and microbial degrading enzymes as the response value. Table 1 also includes the scoring function values calculated from the interaction between the PAEs and the soil microbial complex degrading enzymes (TVSP). SYBYL-X was used to construct S-3D-QSAR using the TVSP scoring function value as the response value.

#### 3.1.2. Evaluation of the 3D-QSAR Model to Study the Comprehensive Effect of Microbial Degradability of PAEs Associated with the Sewage Treatment Process

The evaluation parameters corresponding to the W-3D-QSAR model are presented in Table 2. The optimal principal component n for the W-3D-QSAR model is 8, and the cross-validation coefficient q2 is 0.76. The value indicates that the model has a good predictive ability [39]. The non-cross validation coefficient R2 is 0.999, the standard deviation SEE is 0.009, and the F test value is 1159.179, indicating that the model has good fitting ability and stability. Overfitting was not observed [37]. The standard error obtained post cross-validation (SEP) for the experimental and predicted values was 0.162. The cross-test coefficient corresponding to the external prediction set r2pred was 0.646, indicating high external prediction ability [38]. The contributions of the stereo (S) and electrostatic (E) fields to the combined effect of degradability were 76.5 and 23.5%, respectively, indicating that both spatial and electrical products had an impact on the microbial degradability of PAEs in the sewage treatment process. The spatial outcome was moderately significant [40]. Table 2 shows the parameters of the S-3D-QSAR model, and the evaluation of various parameters reveals that the constructed S-3D-QSAR model meets the model construction requirements.

### 3.2. Molecular Design and Characterization of Alternatives of Readily Biodegradable PAEs Associated with the Sewage Treatment Process

#### 3.2.1. Molecular Design Obtained Based on the 3D-QSAR Models for the Readily Biodegradable Substitutes of PAEs 

**Figure 1:**
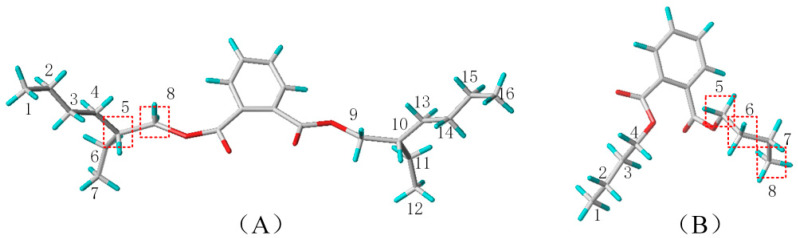
Schematic diagram of the stereological structure and modification sites of DEHP (**A**) and DBP (**B**) molecules.

**Figure 2:**
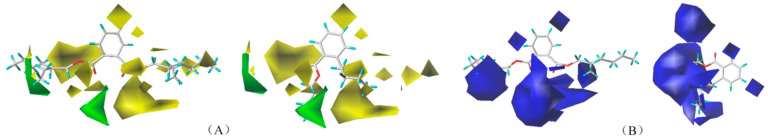
Three-dimensional isopotential maps corresponding to the stereo (**A**) and electrostatic (**B**) fields of the CoMFA model describing the DEHP and DBP molecules.

DEHP and DBP molecules were detected in sewage in high concentrations, and these were used as target molecules (Figure 1) [41]. The CoMFA-based three-dimensional equipotential diagram of the DEHP and DBP W-3D-QSAR models was examined (Figure 2). The molecular stereoscopic field isopotential diagram was created, and the yellow area indicated that increasing the volume of the substituent groups in this area improved the biodegradability of the PAE molecules [42]. In the isophobic diagram of the molecular electrostatic field, the red region overlaps with the blue area. The blue area indicates that an increase in the positive electricity of the substituent group in this region promotes the biodegradability of PAEs [43].

As can be seen from the model molecular stereo field isopotential diagram (Figure 2), the distribution of yellow areas at the ends of sites 3, 5, and 8 of the DEHP molecule and sites 5, 6, 7, and 8 of the DBP molecule indicate that an increase in the volume of the substituent groups around the substitutions mentioned above facilitates the microbial degradability of the PAE molecules during water treatment. The presence of blue areas at the ends of DEHP sites 5, 6, and 8 and DBP sites 3, 5, 6, and 8 indicates that the presence of positively charged groups at these sites promotes the microbial degradability of the PAE molecules during water treatment. In summary, to improve the biodegradability of formate molecules, -COOH (A), -CH2OH (B), -CH2CH3 (C), -CH2COOH (D), -CH2CH2OH (E), -CH2CH2CH3 (F), -CH2CH2CH2CH3 (G), -CH2CH2CH2OH (H), -CH2CH2CH2CH2CH2CH3 (I), -CH2CH2CH2CH2OH (J), -CH2CH2CH2CH2CH2OH (K), -CH2CH2CH2CH2CH2CH3 (L), -CH2CH2OHCH2CH2OH (M), and -CH2CH2CH2CH2OH (N), a total of 14 substituents were selected to perform mono-substitution, di-substitution, and tri-substitution reactions on the target molecules. A total of 38 alternative molecules were designed post substitution, as shown in Table 3. These included 19 alternatives to DBP and 19 alternatives to DEHP (Table S1).

Among the DBP substitutes that meet the increased biodegradability requirement, single-substituted molecules accounted for 21.05% (biodegradability increase: 0.34–0.85%), double-substituted molecules accounted for 52.63% (biodegradability increase: 0.17–41.98%), and triple-substituted molecules accounted for 26.32% (biodegradability increase: 2.22–21.84%) of the total molecules. All of the DEHP substitutes that can meet the biodegradability requirement are double-substituted (biodegradability increase: 0.18–17.46%). The percentage of mono-, di-, and tri-substituted molecules and the increase in microbial degradability during water treatment revealed that the rate of substituted molecules that could meet the increased degradability requirement during water treatment (following the modification of PAEs by mono- and di-substitution) was lower than that achieved for di-substituted molecules. The microbial degradability was not significantly enhanced. The molecular biodegradation effect of PAE substitutes subjected to single substitution processes was the worst, and the increase in biodegradability was ≤1.00%. When PAE molecules are modified under conditions of double substitution, the number of alternative molecules that can meet the requirement is relatively large. The improvement of microbial biodegradability is significant when the -CH3 unit present at sites 5 and 8 of DBP was replaced by -CH2CH2CH2CH2CH3, and under these conditions, the biodegradability of DBP was increased by 41.98%. Therefore, when designing degradable PAE substitutes, priority should be given to double substitution to ensure that the designed substitute molecules significantly improve degradability and design rate.

#### 3.2.2. Aerobic/Anaerobic Biodegradability, Endocrine Disturbance, and Functional Evaluation of PAE Substitutes

To further verify that the PAE alternatives designed by us are readily biodegradable under both aerobic and anaerobic conditions, firstly, the degradation enzymes secreted by aerobic bacteria (1P8O, 1HZV, and 7CUZ) and the degradation enzymes secreted by anaerobic bacteria (2IOF, 1NIC, and 3FMX) were used for protein–protein docking through Discovery Studio 4.0. Aerobic biodegradable complex protein (AEDECP) and anaerobic biodegradable complex protein (ANDECP) were obtained, and the scoring function values were obtained by interacting 38 designed PAEs with the two biodegradable complex proteins [42]. The changes in the molecular scoring function values (before and after molecular modification) occurring post docking of the alternative molecules with AEDECP were analyzed. Thirteen DBP alternatives (3.64–30.34%) and nineteen DEHP alternatives were easily degraded (1.22–58.91%). Following the docking of PAE alternative molecules with ANDECP, 11 DBP alternatives (0.41–41.23%) and all DEHP alternatives were easily degraded (6.78–95.39%). This indicated that the PAE substitutes designed were easily degraded under aerobic and anaerobic conditions [44]. Aerobic microorganisms are better at degrading PAEs than anaerobic microorganisms under sewage treatment conditions [11]. The biodegradability of the PAE substitutes (recorded under anaerobic conditions) designed by us is significantly greater than that achieved in the presence of aerobic microorganisms. This can compensate (to a certain extent) for the poor degradation effect exerted by anaerobic microorganisms during the wastewater treatment process. Under these conditions, the biodegradation efficiency of the PAEs is improved as a whole during the wastewater treatment process.

The T-3D-QSAR model constructed by Han et al. was used to predict the molecular toxicity of the PAE substitutes, and it was expected that the molecular endocrine-disrupting toxicity of the PAE substitutes would not change significantly (within 10%) [45]. In addition, the energy of the stable molecular configurations was calculated using the DFT technique, and the molecular functions corresponding to the designed PAE substitutes were tested [46]. The positive frequency values of 10 DBP molecules and their substitutes were greater than 0, and positive frequency values of 15 DEHP molecules and their substitutes were greater than 0. When the molecule’s positive frequency value is greater than 0, the molecular energy is locally the lowest in a particular dimension, and the molecule can exist stably under these conditions [47]. The rate of change of the energy gap for four DBP and five DEHP substitutes was less than 10. The energy gap value represents the degree of insulation. The larger the energy gap, the stronger the insulation, that is, the more stable the molecular structure [47]. Analysis of the above principles reveals that the functions of the PAE substitutes designed by us do not decrease on the whole, and the molecules can exist stably in the environment.

After screening, the degradable, less toxic, functional PAE substitutes were obtained: DEHP-10, DEHP-12, DBP-5, and DBP-7 (Table 4).

#### 3.2.3. Analysis of the Mechanism of Differential Microbial Degradation before and after Molecular Modification of PAEs

**Figure 3:**
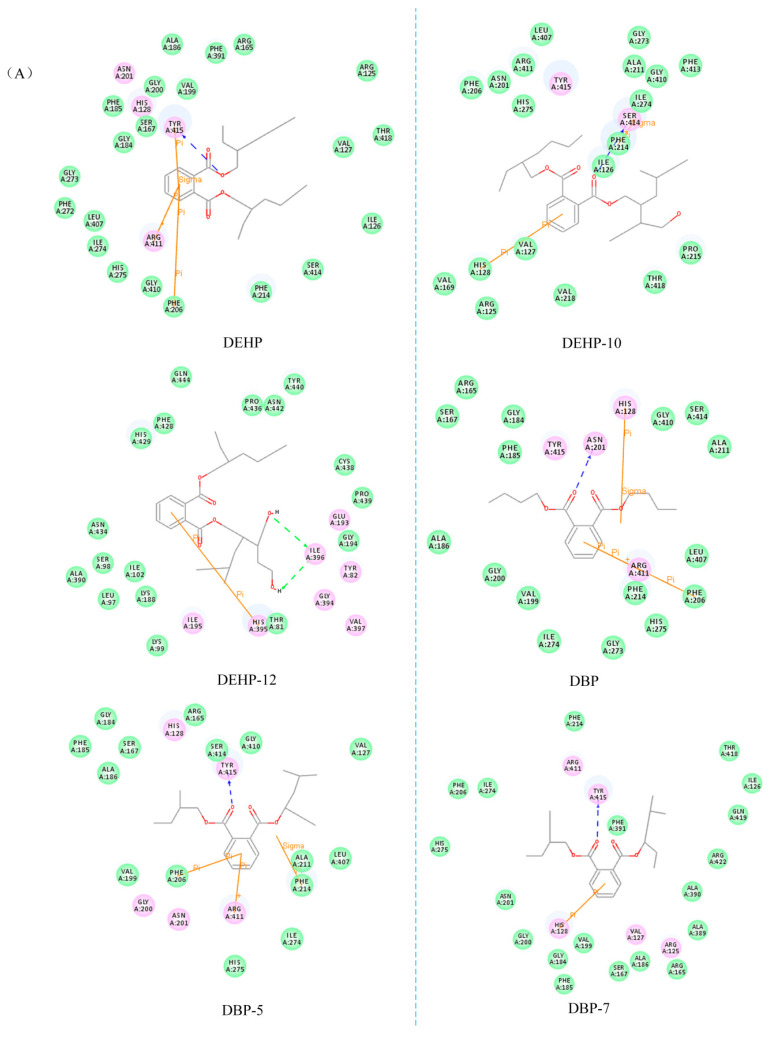

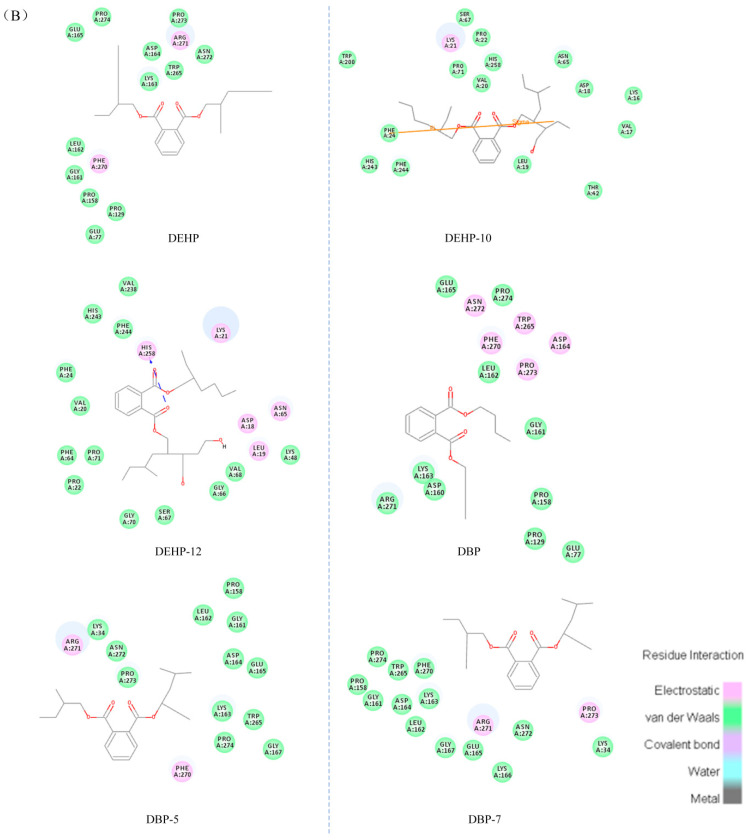
Amino acid residue map of the PAE substitutes binding to aerobic bacteria (**A**)/anaerobic bacteria (**B**) degradable complex proteins.

The biodegradability of the designed DEHP alternatives is significantly improved compared to that of the DBP alternatives. Therefore, the distribution characteristics of the amino acid residues around the molecular docking complexes were used to analyze the differences in molecular biodegradation before and after the modification of PAEs. The results provide theoretical guidance for the design of PAE substitutes. The PAE molecules were docked with AEDECP and ANDECP before and after modification, respectively [42], and the distribution of the amino acid residues around the complex is shown in Figure 3. When DEHP-10 molecules bind to AEDECP and ANDECP, they form Π-Π bonds with multiple hydrophobic amino acids, unlike DEHP molecules. Therefore, the volume of the molecules exposed to solvents is reduced [48]. The hydroxyl group substituent at site 5 of the DEHP-10 molecule is a polar group with solvent affinity [49]. Therefore, the interaction force between DEHP-10 molecules and polar amino acids is strengthened, and the formation of a hydrophilic environment promotes the binding of molecules to complex proteins [50,51]. When DEHP-12 binds to AEDECP and ANDECP, hydrogen bonds and Π–Π bonds are formed between the hydroxyl groups of the substituent groups at site 5 of DEHP-12 and the surrounding amino acids. Therefore, the interaction between the polar amino acids and the molecules is robust [52]. This promotes the binding with complex proteins. These results indicate that the molecular biodegradability of the DEHP substitute increases significantly (40.97–93.72%) during aerobic and anaerobic biodegradation. This can be attributed primarily to interactions between molecules and amino acids. During the binding of DEHP substitutes to complex proteins, numerous Π–Π bonds are formed. Moreover, the substituent groups in the DEHP substitute molecules are primarily polar hydroxyl groups that exhibit solvent affinity. The number of polar amino acids around the molecules accounts is high. This promotes mutual binding between the DEHP substitute molecules and the complex proteins. 

DBP-5 molecules bind to AEDECP and ANDECP, and the substituent ethyl group at the molecular site 6 of DBP-5 is a non-polar group that can form Π-Π bonds with PHE and ARG to improve the binding ability of the system and promote the degradation of complex proteins [49]. However, the strength of the electrostatic interactions between the DBP-5 molecule and the surrounding amino acids is reduced, and the strength of the van der Waals force is increased. The van der Waals force is an attraction between the molecules, and the hydrogen bond is the binding force of the H atom and other atoms. The hydrogen bond is stronger than the van der Waals force [53,54]. The binding ability of the DBP-5 molecule with the degradable complex protein improved, but not significantly. DBP-7 interacts with AEDECP and ANDECP, and the ethyl group at the molecular site 6 of DBP-7 is non-polar. The number of hydrophilic amino acids surrounding DBP-7 is greater than that surrounding DBP. Nonetheless, the number of interacting bonds between the DBP-7 molecule and the surrounding amino acids decreased, and hydrogen bond interactions were less than van der Waals interactions. Therefore, the binding ability of the DBP-7 molecule increased to a certain extent, but the change was not significant.

A comparison of the degree of biodegradability of the DEHP and DBP substitutes revealed that the nature of the different substituent groups on the PAE substitutes dictated the differences in their microbial degradability. The ability to form hydrogen and Π-Π bonds with surrounding amino acids dictated the binding efficiency of the microbial degradation enzymes. Therefore, polar substituent groups should be selected while designing PAE substitute molecules. This is due to the fact that polar substituent groups are solvent-friendly and can easily form hydrogen and Π-Π bonds with the amino acids around them. This improves the binding efficiency of the PAE substitutes, resulting in a greater degree of microbia degradability.

### 3.3. Evaluation and Mechanism Analysis of the Process of Photodegradation and Microbial Degradation Potential of DEHP Substitutes in the Soil Environment

#### 3.3.1. Soil Environmental Photodegradation and Microbial Degradation Pathways (before and after DEHP Molecular Modification)

**Figure 4:**
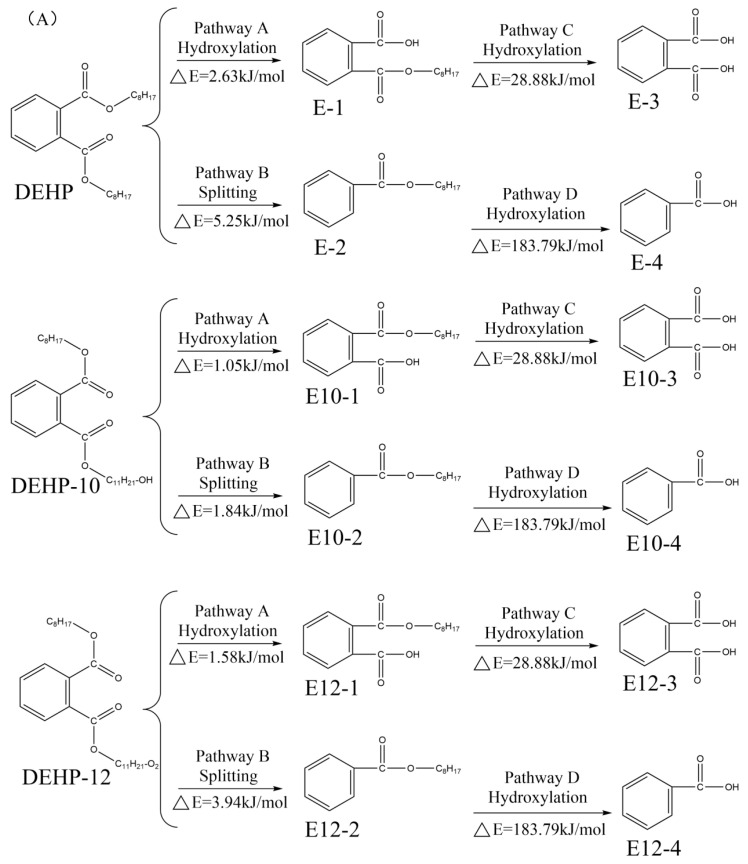

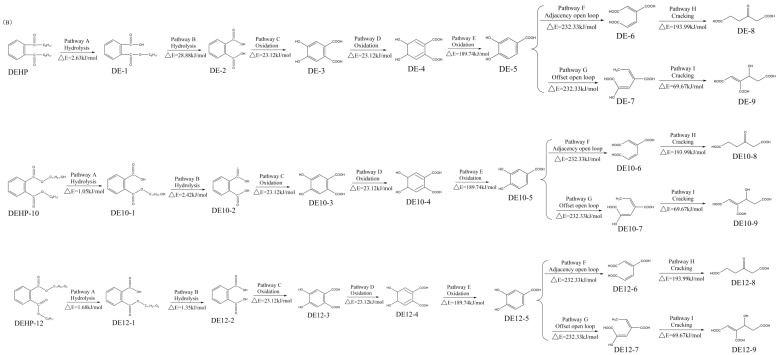
Photodegradation pathway (**A**)/Microbial degradation pathway (**B**) associated with DEHP and its substitutes in the soil environment.

The PAEs that cannot be removed during sewage treatment can enter the soil environment through secondary biochemical effluents [13], thus, affecting the soil farming environment and crop growth conditions. These can enter the human body through the food chain, posing a threat to human health [15]. Improving the degradability of PAEs in the soil is, therefore, critical. According to previous research, photodegradation and microbial degradation are the primary mechanisms for PAE degradation in soil [20,55]. Furthermore, two kinds of DEHP substitutes (DEHP-10 and DEHP-12), characterized by high biodegradability, were designed and screened as examples to verify whether they are characterized by high photodegradation and microbial degradation potential in the soil environment. The DEHP molecules are cracked and hydrolyzed in the soil during photodegradation [56]. DEHP molecules are hydroxylated and cleaved by light to form 2-carboxybenzaldehyde (E-1) and octyl benzoate (E-2), respectively. The C-O bonds in E-1 and E-2 are broken to form phthalic acid (E-3) and benzoic acid (E-4), and the compounds are finally mineralized to CO2 and H2O, resulting in linear degradation [57]. The photodegradation pathway of the DEHP molecule can be used to infer the photodegradation pathway of the substitute molecule [55], as shown in Figure 4. The hydroxylation and cleavage reactions of DEHP substitutes also occur under the action of light. During the degradation of DEHP substitutes, -OH attacks the side chain and causes the C-O bond to break. This results in the formation of phthalic acid and benzoic acid, which are finally mineralized into CO2 and H2O following cleavage.

The primary microorganisms associated with microbial degradation in soil include Geobacillus, Pseudomonas adaceae, Trichoderma reesei, and Streptomyces [58], and the primary degradation mode involves the synergistic degradation of the compound under the action of mixed strains [10]. DEHP undergoes hydroxylation, oxidation, ortho-ring opening, and meta-ring opening cleavage reactions [59]. First, the DEHP molecule is hydroxylated to 2-carboxybenzaldehyde (DE-1). The C-O bond in DE-1 is then broken to form phthalic acid (DE-2), which is then oxidized to protocatechuic acid (DE-5). Under epicyclic conditions, DE-5 is cleaved into acetyl-CoA, succinic acid (DE-8), pyruvic acid, and oxaloacetic acid (DE-9). It then enters the tricarboxylic acid cycle, where it is oxidized to CO2 and H2O [7]. The molecular microbial degradation path of DEHP was inferred from the molecular microbial degradation path of the substitute, as shown in Figure 4. Hydroxylation, oxidation, ortho ring opening, and meta-ring opening cracking reactions also occurred during the microbial degradation of the DEHP substitutes. During the degradation of the DEHP substitute molecules, -OH attacks the side chain and causes the C-O bond to break to form benzoic acid ester products. These are then oxidized and cracked to produce acetyl-CoA, succinic acid, pyruvate acid, and oxaloacetate. Finally, CO2 and H2O are produced under conditions of oxidation.

#### 3.3.2. Evaluation of the Extent of Photodegradation and Microbial Degradation Responses Produced and Product Toxicity in Soils before and after Molecular Modification by DEHP

The photodegradation and microbial degradation processes in soil associated with DEHP substitutes, as well as the product’s toxicity, were investigated. To calculate or predict the reaction energy barrier and endocrine-disrupting toxicity associated with the photodegradation and microbial degradation pathways of the DEHP substitutes, the DFT technique, and the H-QSAR model were used. The greater the extent of molecular photodegradation and microbial degradation of DEHP substitutes, the lower the reaction energy barrier [60]. It was calculated that the E10-1 reaction energy barrier (ΔE = 1.05 kJ/mol) corresponding to the DEHP-10 molecular pathway A (associated with soil photodegradation) was 60.08% lower than the E-1 reaction energy barrier (ΔE = 2.63 kJ/mol) recorded before modification. The E10-2 reaction energy barrier in path B (ΔE = 1.84 kJ/mol) was 64.95% lower than the E-2 reaction energy barrier achieved before modification (ΔE = 5.25 kJ/mol). The E12-1 reaction energy barrier (ΔE = 1.58 kJ/mol) associated with the DEHP-12 molecular pathway A was 39.92% lower than the E-1 reaction energy barrier achieved before modification. The E12-2 reaction energy barrier in path B (ΔE = 3.94 kJ/mol) was 24.95% lower than the E-2 reaction energy barrier achieved before modification. This indicates that the designed DEHP alternatives were more photodegradable in soil than the DEHP molecules. The toxicity of the photodegradation intermediates does not change because the same degradation intermediates are formed when DEHP substitutes and DEHP are degraded.

The reaction energy barrier corresponding to the soil microbial degradation pathway for the degradation of the DEHP substitute was calculated. It was observed that the DE10-1 reaction energy barrier (ΔE = 1.05 kJ/mol) in path A corresponding to the DEHP-10 alternative molecule was 60.08% lower compared to that of the DE-1 molecule before modification (ΔE = 2.63 kJ/mol). The energy barrier for the DE10-2 reaction in path B (ΔE = 2.42 kJ/mol) was 91.62% lower than the energy barrier recorded for the DE-2 reaction before modification (ΔE = 28.88 kJ/mol). The DE12-1 reaction energy barrier (ΔE = 1.68 kJ/mol) for the DEHP-12 molecular pathway A was 36.12% lower than that recorded for the DE-1 reaction energy barrier before the modification. The energy barrier for the DE12-2 reactions in path B (ΔE = 1.35 kJ/mol) was 95.33% lower than for the DE-2 reaction before the modification. This indicates that it is easier to degrade the designed DEHP alternative in the presence of microorganisms compared to the DEHP molecules. Two different intermediates (DE10-1 and DE12-1) were formed during the process of microbial degradation of the DEHP replacement molecule in soils. The T-QSAR model was used to predict the toxicity values of the DE10-1 and DE12-1 molecules [20]. The results showed that DE10-1 and DE12-1 were 4.85 and 17.26% more toxic than DE-1, respectively. Analysis revealed that DE10-1 and DE12-1 altered their toxicity by lengthening the carbon chain following substitution reactions and changing molecular weights [61]. In contrast, the intermediate DE12-1 in pathway A bears an aldehyde moiety that can covalently cross-link with proteins, nucleic acids, and lipids in organisms [62]. This results in protein glycosylation or carbonylation [63,64] and the upregulation of the DE12-1 endocrine-disrupting toxicity [7]. Therefore, given the significant increase in the toxicity value of DE12-1, an intermediate produced during the soil microbial degradation process of the DEHP-12 molecule, it is not recommended as a choice for the DEHP molecule.

#### 3.3.3. Analysis of the DEHP Substitutes Suitable for Sewage Treatment and the Soil Microbial Degradation Mechanism

**Figure 5:**
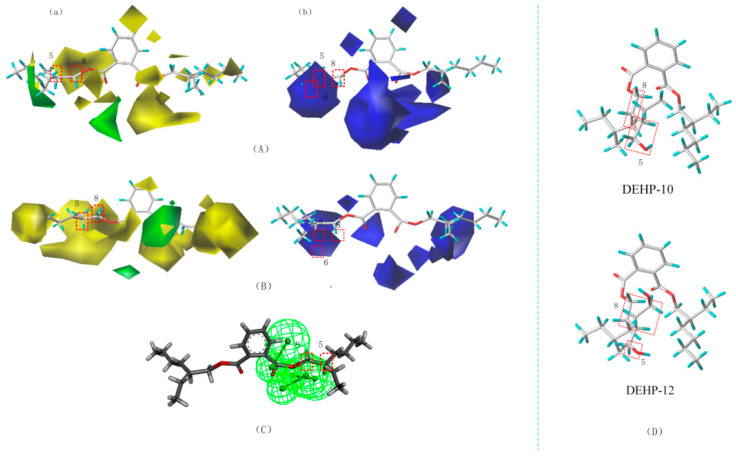
W-3D-QSAR model (**A**), S-3D-QSAR model (**B**), (stereo field a, electrostatic field b), T-QSAR model (**C**), and schematic diagram of the molecular modification sites of DEHP substitutes (**D**).

To further validate that DEHP-10 and DEHP-12 molecules follow easy degradation mechanisms, difference analysis methods were used from the perspective of the three-dimensional equipotential diagram of the W-3D-QSAR, S-3D-QSAR, and T-QSAR models (Figure 5). As can be seen from Figure 5, analysis of the stereo field isopotential maps corresponding to the W-3D-QSAR and S-3D-QSAR models reveals that yellow regions are distributed at the ends of the 5 and 8 methyl groups of the DEHP molecule. It indicates that the groups located at these sites are replaced by larger groups, which promotes the degradation of molecules during the sewage treatment process in the soil environment [65]. Blue areas are distributed at the ends of the 5, 6, and 8 methyl groups of the DEHP in the isopotential diagram of the model electrostatic field. These findings indicated that the substitution of positively charged groups at these sites aided in the degradation of molecules during the sewage treatment process in the soil environment [43]. Analysis of the superimposed plot of the T-QSAR model reveals that the DEHP molecule binds to three hydrogen-bonded receptors at sites 5 and 8, suggesting that substitution at these sites can alter the endocrine-disrupting properties of the molecule [66]. Therefore, common substitution sites (5 and 8) are present in the three-dimensional equipotential map of the W-3D-QSAR, S-3D-QSAR, and T-QSAR models, and the same substituent selection rules are used to improve the degradability of the PAEs.

The DEHP-10 and DEHP-12 molecules screened in this paper were designed with a double substitution at sites 5 and 8, and the carbon chain length was extended. The substituent groups were changed to improve the microbial degradability during sewage treatment and ensure their toxicity did not increase [65]. When the DEHP-10 molecule was doubly substituted at sites 5 and 8, the -CH3 group at site 5 was replaced by the -CH2CH2OH group, and the -CH3 group at site 8 was replaced by the -CH2CH2CH3 group. The DEHP-12 molecule was doubly substituted at sites 5 and 8, whereas the -CH3 group at site 5 replaced by the -CH2OH group and the -CH3 group at site 8 replaced by the -CH2CH2CH2CH2CH2OH group. The modification pathways associated with the DEHP-10 and DEHP-12 molecules are consistent with the common distribution characteristics of the 3D isopotential maps of the W-QSAR, S-QSAR, and T-QSAR models. Therefore, the PAE replacements designed by us can satisfy both the biodegradability conditions of the sewage treatment process and the soil environment conditions while safeguarding against elevated toxicity.

## 4. Conclusions

This paper proposed a molecular screening system for easily degradable substitutes based on the prediction of degradability models for water treatment processes and soil environment, and the degradation pathways were constructed for PAE molecules. Based on the W-3D-QSAR, S-3D-QSAR, and T-3D-QSAR models, two alternative molecules for PAE with unchanged endocrine disruptor toxicity and easy degradation were successfully designed. The molecular degradability of the molecules was investigated during the sewage treatment process and in the soil environment. It was discovered that the molecular degradability of the PAE substitutes screened in this paper increased by 51.31–97.13% during sewage treatment and 33.18–62.94% in the soil environment. The distribution characteristics of the amino acid residues around the PAE molecules and docking complexes (before and after modification) were analyzed, and the coupling conditions were designed based on the 3D-QSAR model. Finally, the results provide theoretical guidance for the molecular design of easily degradable PAE substitutes to solve the problem of molecular persistence of PAEs in nature.

## Figures and Tables

**Table 1:** Comprehensive effect value of PAEs and microbial degradability associated with the water treatment process, and the scoring function value of PAEs and the soil microbial complex degrading enzyme.

No.	Compounds	Docking Scores						Score	Docking Scores
		1HZV	1P80	7CUZ	1NIC	2IOF	3FMX		TVSP
1	Butyl benzyl phthalate (BBP)	60.75	92.62	57.81	101.16	102.90	67.45	0.69	78.48
2	Diallyl phthalate (DAP)	56.31	71.36	52.66	78.92	76.78	59.60	0.40	63.02
3	Dibutyl phthalate (DBP)	66.41	82.54	64.56	101.04	85.22	75.90	0.66	84.09
4	Diethyl phthalate (DEP)	43.52	60.46	50.31	83.82	68.76	49.11	0.32	59.74
5	Dihexyl phthalate (DHP)	76.02	106.04	78.84	62.19	101.86	78.31	0.60	104.63
6	Diisobutyl phthalate (DIBP)	68.90	83.48	54.34	89.15	81.14	73.42	0.56	70.46
7	Diisoheptyl phthalate (DIHP)	82.08	52.24	90.46	55.95	105.16	81.75	0.53	106.61
8	Bis (4-methylpentyl) phthalate (DIHXP)	72.91	99.31	85.17	40.05	99.77	70.39	0.44	81.29
9	Diisopentyl phthalate (DIPP)	73.03	90.29	62.19	99.48	93.98	64.95	0.67	80.99
10	Diisopropylo-phthalate (DIPRP)	48.66	65.12	48.70	81.83	69.97	49.44	0.33	65.17
11	Bis (2-methoxyethyl) phthalate (DMEP)	61.98	78.64	63.56	84.66	86.95	68.18	0.53	86.18
12	Dimethyl phthalate (DMP)	52.85	52.25	47.59	73.86	61.86	38.89	0.21	52.74
13	Di-n-octylo-phthalate (DNOP)	90.08	123.21	99.19	83.44	100.47	79.24	0.82	83.55
14	Di-N-pentyl phthalate (DPP)	74.17	97.39	66.60	50.16	97.95	68.34	0.44	90.28
15	Dipropyl phtalate (DPRP)	57.54	72.99	56.65	71.04	76.31	65.00	0.38	69.68
16	Diisotridecyl phthalate (DTDP)	100.64	101.47	109.35	90.05	91.38	71.89	0.81	98.39
17	Diundecyl phthalate (DUP)	99.87	131.46	105.18	72.27	92.33	75.26	0.76	102.92
18	Bis (2-ethylhexyl) phthalate (DEHP)	86.29	103.40	88.43	57.21	100.61	83.03	0.61	80.77
19	Dibenzyl phthalate (DBZP)	74.74	100.09	78.57	60.45	99.27	61.87	0.69	76.01
20	Dicyclohexyl phthalate (DCHP)	68.10	87.82	59.03	64.61	85.92	42.15	0.40	73.77
21	Diisodecyl phthalate (DIDP)	103.39	128.09	82.34	76.60	105.08	101.72	0.66	122.40
22	Diisononyl phthalate (DINP)	87.46	125.33	79.90	50.32	100.11	93.76	0.32	86.11
23	Diisooctyl phthalate (DIOP)	82.03	126.98	73.58	76.60	100.42	86.34	0.59	84.44
24	Didecyl phthalate (DNDP)	112.76	128.69	81.37	102.99	101.64	100.05	0.56	111.87
25	Dinonyl phthalate (DNP)	97.61	126.02	88.44	80.63	100.48	110.94	0.53	117.49
26	Dioctyl Phthalate (DOP)	86.30	123.21	74.87	83.44	103.36	87.53	0.44	102.07
27	Diphenyl phthalate (DPHP)	46.80	81.72	87.88	90.44	71.16	31.56	0.67	55.16
28	Octyldecyl phthalate (ODP)	89.10	127.87	96.76	92.52	100.45	96.62	0.33	101.73

**Table 2:** Parameters associated with the PAEs of the W-3D-QSAR and S-3D-QSAR models.

3D-QSAR Model	n	q^2^	r^2^	F	SEE	*r* ^2^_pred_	SEP	S	E
Sewage treatment process Comprehensive effect of microbial degradability	8	0.76	0.999	1159.179	0.009	0.646	0.162	76.50%	23.50%
Soil microbial degradability	9	0.88	1.000	24128.603	0.188	0.957	15.719	65.48%	32.52%

**Table 3:** Prediction and change rate of molecular biodegradation of DBP and DEHP substitutes.

Molecular	Substitution Sites and Substituents	Predictive Value	Change Rate	Molecular	Substitution Sites and Substituents	Predictive Value	Change Rate
DBP	-	0.586	-	DEHP	-	0.567	-
DBP-1	5-F-6-C	0.614	4.78%	DEHP-1	5-B-8-B	0.617	8.82%
DBP-2	5-G-6-C-8-C	0.643	9.73%	DEHP-2	5-C-8-D	0.59	4.06%
DBP-3	5-C-6-C	0.596	1.71%	DEHP-3	5-B-8-K	0.568	0.18%
DBP-4	5-F	0.591	0.85%	DEHP-4	5-E-8-L	0.569	0.35%
DBP-5	6-C	0.588	0.34%	DEHP-5	5-E-8M	0.618	8.99%
DBP-6	5-G-6-F-8-C	0.679	15.87%	DEHP-6	5-E-8-E	0.57	0.53%
DBP-7	5-C	0.589	0.51%	DEHP-7	5-B-8-F	0.604	6.53%
DBP-8	5-I-8-C	0.636	8.53%	DEHP-8	5-C-8-G	0.574	1.23%
DBP-9	8-G	0.591	0.85%	DEHP-9	5-H-8-C	0.572	0.88%
DBP-10	5-I-8-I	0.832	41.98%	DEHP-10	5-E-8-E	0.633	11.64%
DBP-11	5-F-6-E	0.595	1.54%	DEHP-11	5-B-8-G	0.613	8.11%
DBP-12	5-C-6-F	0.605	3.24%	DEHP-12	5-H-8-E	0.629	10.93%
DBP-13	5-G-6-F	0.617	5.29%	DEHP-13	5-E-8-H	0.63	11.11%
DBP-14	5-F-6-C-8-F	0.666	13.65%	DEHP-14	5-E-8-J	0.647	14.11%
DBP-15	6-C-8-C	0.587	0.17%	DEHP-15	5-B-8-N	0.666	17.46%
DBP-16	5-G-6-F-8-C	0.714	21.84%	DEHP-16	5-E-8-K	0.648	14.29%
DBP-17	5-B-8-C	0.589	0.51%	DEHP-17	5-B-8-K	0.621	9.52%
DBP-18	5-C-6-C-8-C	0.599	2.22%	DEHP-18	5-C-8-K	0.573	1.06%
DBP-19	5-G-8-A	0.632	7.85%	DEHP-19	5-C-8-L	0.576	1.59%

**Table 4:** Molecular degradability, toxicity, and functional verification of PAE substitutes.

Molecular	The Scoring Function Value of Docking with AEDECP	Change Rate	The Scoring Function Value of Docking with ANDECP	Change Rate	Toxicity	Change Rate	Frequency	Energy Gap
DEHP	84.91	-	49.53	-	0.66	-	8.49	0.20
DEHP-10	119.70	40.97%	79.59	60.69%	0.64	−3.03%	6.16	0.20
DEHP-12	121.00	42.50%	95.95	93.72%	0.67	1.52%	4.47	0.20
DBP	99.90	-	64.20	-	0.53	-	10.70	0.20
DBP-5	108.91	9.02%	68.35	6.46%	0.58	9.43%	14.32	0.21
DBP-7	107.11	7.22%	64.78	0.90%	0.56	5.67%	9.78	0.21

## Data Availability

No data was reported in this study.

## Supplementary Materials

The following supporting information can be downloaded at: https://www.mdpi.com/article/10.3390/toxics10120783/s1, Figure S1: The docking site of proteins; Figure S2: Molecular structure diagram of PAEs; Figure S3: The structure of proteins; Table S1: Molecular structure of DBP and DEHP substitutes.

## References

[1] González-Sálamo J., González-Curbelo M.Á., Socas-Rodríguez B., Hernández-Borges j., Rodríguez-Delgado M.Á. (2018). Determination of phthalic acid esters in water samples by hollow fiber liquid-phase microextraction prior to gas chromatography tandem mass spectrometry. Chemosphere.

[2] Kaewlaoyoong A., Vu C.T., Lin C., Liao C.S., Chen J.R. (2018). Occurrence of phthalate esters around the major plastic industrial area in southern Taiwan. Environ. Earth Sci..

[3] Tan S., Wang D., Chi Z., Li W., Shan Y. (2017). Study on the interaction between typical phthalic acid esters (PAEs) and human haemoglobin (hHb) by molecular docking. Environ. Toxicol. Pharmacol..

[4] Waller C.L., Griffiths H.J., Waluda C.M., Thorpe S.E., Loaiza L., Moreno B., Pacherres C.O., Hughes K.A. (2017). Microplastics in the Antarctic marine system: An emerging area of research. Sci. Total Environ..

[5] Luo H., Liu C., He D., Xu J., Sun J., Li J., Pan X. (2022). Environmental behaviors of microplastics in aquatic systems: A systematic review on degradation, adsorption, toxicity and biofilm under aging conditions. J. Hazard. Mater..

[6] Isobe A., Iwasaki S., Uchida K., Tokai T. (2019). Abundance of non-conservative microplastics in the upper ocean from 1957 to 2066. Nat. Commun..

[7] Chen F., Chen Y., Chen C., Feng L., Dong Y., Chen J., Lan J., Hou H. (2021). High-efficiency degradation of phthalic acid esters (PAEs) by Pseudarthrobacter defluvii E5: Performance, degradative pathway, and key genes. Sci. Total Environ..

[8] Eleonora G., Antoni S., Silvia T., Caterina F. (2018). Microplastic in marine organism: Environmental and toxicological effects. Environ. Toxicol. Pharmacol..

[9] Das M.T., Kumar S.S., Ghosh P., Shah G., Malyan S.K., Bajar S., Thakur I.S., Singh L. (2021). Remediation strategies for mitigation of phthalate pollution: Challenges and future perspectives. J. Hazard. Mater..

[10] Becky M.I., Anbalagan K., Magesh K.M. (2022). Phthalates removal from wastewater by different methods–a review. Water Sci. Technol..

[11] Tran H.T., Lin C., Bui X.T., Nguyen M.K., Cao N.D.T., Mukhtar H., Hoang H.G., Varjani S., Ngo H.H., Nghiem L.D. (2022). Phthalates in the environment: Characteristics, fate and transport, and advanced wastewater treatment technologies. Bioresour. Technol..

[12] Perpetuo E.A., Silva E.C.N.D., Karolski B., Nascimento C.A.O.D. (2020). Biodegradation of diethyl-phthalate (DEP) by halotolerant bacteria isolated from an estuarine environment. Biodegradation.

[13] Wei L., Li Z., Sun J., Zhu L. (2020). Pollution characteristics and health risk assessment of phthalate esters in agricultural soil and vegetables in the Yangtze River Delta of China. Sci. Total Environ..

[14] Liu W., Zhang J., Liu H., Guo X., Zhang X., Yao X., Cao Z., Zhang T. (2021). A review of the removal of microplastics in global wastewater treatment plants: Characteristics and mechanisms. Environ. Int..

[15] Wang Y., Zhu H., Kannan K. (2019). A review of biomonitoring of phthalate exposures. Toxics.

[16] Lü H., Mo C.H., Zhao H.M., Xiang L., Katsoyiannis A., Li Y.W., Cai Q.Y., Wong M.H. (2018). Soil contamination and sources of phthalates and its health risk in China: A review. Environ. Res..

[17] He M., Yang T., Yang Z., Zhou H., Wei S. (2018). Current state, distribution, and sources of phthalate esters and organophosphate esters in soils of the three gorges reservoir region, China. Arch. Environ. Contam. Toxicol..

[18] Wang D., Xi Y., Shi X.Y., Zhong Y.J., Guo C.L., Han Y.N., Li F.M. (2021). Effect of plastic film mulching and film residues on phthalate esters concentrations in soil and plants, and its risk assessment. Environ. Pollut..

[19] Lee Y.M., Lee J.E., Choe W., Kim T., Lee J.Y., Kho Y., Choi K., Zoh K.D. (2019). Distribution of phthalate esters in air, water, sediments, and fish in the Asan Lake of Korea. Environ. Int..

[20] Sun J., Zhu H., Yang X., Zheng Y., Sun T., Xu H., Meng J., Zhang A. (2022). Carboxylesterase and lipase-catalyzed degradation of phthalate esters in soil and water: Congener structure selectivity and specificity. Environ. Technol. Innov..

[21] Zeng X., Qu R., Feng M., Chen J., Wang L., Wang Z. (2016). Photodegradation of polyfluorinated dibenzo-p-dioxins in organic solvents: Experimental and theoretical studies. Environ. Sci. Technol..

[22] Courtene-Jones W., Quinn B., Gary S.F., Mogg A.O.M., Narayanaswamy B.E. (2017). Microplastic pollution identified in deep-sea water and ingested by benthic invertebrates in the Rockall Trough, North Atlantic Ocean. Environ. Pollut..

[23] Xu X., Meng L., Luo J., Zhang M., Wang Y., Dai Y., Sun C., Wang Z., Yang S., He H. (2021). Self-assembled ultrathin CoO/Bi quantum dots/defective Bi2MoO6 hollow Z-scheme heterojunction for visible light-driven degradation of diazinon in water matrix: Intermediate toxicity and photocatalytic mechanism. Appl. Catal. B-Environ..

[24] Zhou B., Zhao L., Wang Y., Sun Y., Li X., Xu H., Weng L., Pan Z., Yang S., Chang X. (2020). Spatial distribution of phthalate esters and the associated response of enzyme activities and microbial community composition in typical plastic-shed vegetable soils in China. Ecotoxicol. Environ. Saf..

[25] Qi X., Li T., Wang F., Dai Y., Liang W. (2018). Removal efficiency and enzymatic mechanism of dibutyl phthalate (DBP) by constructed wetlands. Environ. Sci. Pollut. Res..

[26] Sarkar J., Dutta A., Chowdhury P.P., Chakraborty J., Dutta T.K. (2020). Characterization of a novel family VIII esterase EstM2 from soil metagenome capable of hydrolyzing estrogenic phthalates. Microb. Cell Factories.

[27] Bhattacharyya M., Basu S., Dhar R., Dutta T.K. (2022). Phthalate hydrolase: Distribution, diversity and molecular evolution. Environ. Microbiol. Rep..

[28] Wang X., Han B., Wu P., Li S., Lv Y., Lu J., Yang Q., Li J., Zhu Y., Zhang Z. (2020). Dibutyl phthalate induces allergic airway inflammation in rats via inhibition of the Nrf2/TSLP/JAK1 pathway. Environ. Pollut..

[29] Hu R., Zhao H., Xu X., Wang Z., Yu K., Shu L., Yan Q., Wu B., Mo C., He Z. (2021). Bacteria-driven phthalic acid ester biodegradation: Current status and emerging opportunities. Environ. Int..

[30] Mesdaghinia A., Azari A., Nodehi R.N., Yaghmaeian K., Bharti A.K., Agarwal S., Gupta V.K., Sharafi K. (2017). Removal of phthalate esters (PAEs) by zeolite/Fe3O4: Investigation on the magnetic adsorption separation, catalytic degradation and toxicity bioassay. J. Mol. Liq..

[31] Fan S., Wang J., Yan Y., Wang J., Jia Y. (2018). Excellent degradation performance of a versatile phthalic acid esters-degrading bacterium and catalytic mechanism of monoalkyl phthalate hydrolase. Int. J. Mol. Sci..

[32] Zhao Y., Hou Y., Li Y. (2020). Multi-directional selective toxicity effects on farmland ecosystems: A novel design of green substitutes for neonicotinoid insecticides. J. Clean. Prod..

[33] Wang X., Xiang W., Wang S., Ge J., Qu R., Wang Z. (2019). Oxidative oligomerization of phenolic endocrine disrupting chemicals mediated by Mn (III)-L complexes and the role of phenoxyl radicals in the enhanced removal: Experimental and theoretical studies. Environ. Sci. Technol..

[34] Zhang H., Zhao C., Na H. (2022). PAEs Derivatives’ Design for Insulation: Integrated In-Silico Methods, Functional Assessment and Environmentally Friendly Molecular Modification. Int. J. Environ. Res. Public Health.

[35] Yu H., Zhang B. (2019). A hybrid MADM algorithm based on attribute weight and utility value for heterogeneous network selection. J. Netw. Syst. Manag..

[36] Li Q., Qiu Y., Li Y. (2020). Molecular design of environment-friendly PAE derivatives based on 3D-QSAR assisted with a comprehensive evaluation method combining toxicity and estrogen activities. Water Air Soil Pollut..

[37] Gu W., Zhao Y., Li Q., Li Y. (2019). Environmentally friendly polychlorinated naphthalenes (PCNs) derivatives designed using 3D-QSAR and screened using molecular docking, density functional theory and health-based risk assessment. J. Hazard. Mater..

[38] Hou Y., Zhao Y., Li Q., Li Y. (2020). Highly biodegradable fluoroquinolone derivatives designed using the 3D-QSAR model and biodegradation pathways analysis. Ecotoxicol. Environ. Saf..

[39] Li X., He W., Zhao Y., Chen B., Zhu Z., Kang Q., Zhang B. (2022). Dermal exposure to synthetic musks: Human health risk assessment, mechanism, and control strategy. Ecotoxicol. Environ. Saf..

[40] Li X., Zhao Y., Chen B., Zhu Z., Kang Q., Husain T., Zhang B. (2022). Inhalation and ingestion of synthetic musks in pregnant women: In silico spontaneous abortion risk evaluation and control. Environ. Int..

[41] Le T.M., Nguyen H.M.N., Nguyen V.K., Nguyenet A.V., Vu N.D., Yen N.T.H., Hoang A.Q., Minh T.B., Kannan K., Tran T.M. (2021). Profiles of phthalic acid esters (PAEs) in bottled water, tap water, lake water, and wastewater samples collected from Hanoi, Vietnam. Sci. Total Environ..

[42] Li X., Gu W., Chen B., Zhu Z., Zhang B. (2021). Functional modification of HHCB: Strategy for obtaining environmentally friendly derivatives. J. Hazard. Mater..

[43] Liu H., Huang H., Xiao X., Zhao Z., Liu C. (2021). Effects of phthalate esters (PAEs) on cell viability and Nrf2 of HepG2 and 3D-QSAR studies. Toxics.

[44] Li Y., Wang J., Yang S., Zhang S. (2021). Occurrence, health risks and soil-air exchange of phthalate acid esters: A case study in plastic film greenhouses of Chongqing, China. Chemosphere.

[45] Han Z., Yang L., Du M., Li Y. (2020). A novel pharmacophore model on PAEs’ estrogen and thyroid hormone activities using the TOPSIS and its application in molecule modification. Environ. Sci. Pollut. Res..

[46] Liao X., Lu R., Xia L., Liu Q., Wang H., Zhao K., Wang Z., Zhao Y. (2022). Density functional theory for electrocatalysis. Energy Environ. Mater..

[47] Abdel-Kader N.S., Abdel-Latif S.A., El-Ansary A.L., Sayed A.G. (2021). Spectroscopic studies, density functional theory calculations, non-linear optical properties, biological activity of 1-hydroxy-4-((4-(N-(pyrimidin-2-yl) sulfamoyl) phenyl) diazenyl)-2-naphthoic acid and its chelates with Nickel (II), Copper (II), Zinc (II) and Palladium (II) metal ions. J. Mol. Struct..

[48] Geng H., Zhang P., Peng Q., Cui J., Hao J., Zeng H. (2022). Principles of Cation−π Interactions for Engineering Mussel-Inspired Functional Materials. Acc. Chem. Res..

[49] Liu J., Hua D., Zhang Y., Japip S., Chung T.S. (2018). Precise molecular sieving architectures with Janus pathways for both polar and nonpolar molecules. Adv. Mater..

[50] Wang W., Liang Y., Jin Y., Zhang J., Su J., Li Q. (2021). E484K mutation in SARS-CoV-2 RBD enhances binding affinity with hACE2 but reduces interactions with neutralizing antibodies and nanobodies: Binding free energy calculation studies. J. Mol. Graph. Model..

[51] Gontrani L. (2018). Choline-amino acid ionic liquids: Past and recent achievements about the structure and properties of these really “green” chemicals. Biophys. Rev..

[52] Shen W., He P., Xiao C., Chen X. (2018). From Antimicrobial Peptides to Antimicrobial Poly (α-amino acid) s. Adv. Healthc. Mater..

[53] Wang J., Zheng H., Zhang S., Li J., Zhu X., Jin H., Xu J. (2021). Improvement of protein emulsion stability through glycosylated black bean protein covalent interaction with (−)-epigallocatechin-3-gallate. RSC Adv..

[54] Kolesov B.A. (2021). Hydrogen bonds: Raman spectroscopic study. Int. J. Mol. Sci..

[55] Feng J., Deng Q., Ni H. (2022). Photodegradation of phthalic acid esters under simulated sunlight: Mechanism, kinetics, and toxicity change. Chemosphere.

[56] Ye Q., Liu C., Wu P., Wu J., Lin L., Li Y., Ahmed Z., Rehman S., Zhu N. (2022). Insights into photocatalytic degradation of phthalate esters over MSnO3 perovskites (M=Mg, Ca): Experiments and density functional theory. J. Environ. Manag..

[57] Mphahlele I.J., Malinga S.P., Dlamini L.N. (2022). Combined biological and photocatalytic degradation of dibutyl phthalate in a simulated wastewater treatment plant. Catalysts.

[58] Du M., Li X., Cai D., Zhao W., Wang J., Li Y. (2022). In-silico study of reducing human health risk of POP residues’ direct (from tea) or indirect exposure (from tea garden soil): Improved rhizosphere microbial degradation, toxicity control, and mechanism analysis. Ecotoxicol. Environ. Saf..

[59] Kaur R., Kumari A., Sharma G., Singh D., Kaur R. (2021). Biodegradation of endocrine disrupting chemicals benzyl butyl phthalate and dimethyl phthalate by Bacillus marisflavi RR014. J. Appl. Microbiol..

[60] Gu W., Zhao Y., Li Q., Li Y. (2020). Plant-microorganism combined remediation of polychlorinated naphthalenes contaminated soils based on molecular directed transformation and Taguchi experimental design-assisted dynamics simulation. J. Hazard. Mater..

[61] Han J., Zhang X. (2018). Evaluating the comparative toxicity of DBP mixtures from different disinfection scenarios: A new approach by combining freeze-drying or rotoevaporation with a marine polychaete bioassay. Environ. Sci. Technol..

[62] Nakamura J., Nakamura M. (2020). DNA-protein crosslink formation by endogenous aldehydes and AP sites. DNA Repair.

[63] Li J., Zhang J., Xu M., Yang Z., Yue S., Zhou W., Gui C., Zhang H., Li S., Wang P.G. (2022). Advances in glycopeptide enrichment methods for the analysis of protein glycosylation over the past decade. J. Sep. Sci..

[64] Gonos E.S., Kapetanou M., Sereikaite J., Bartosz G., Naparlo K., Grzesik M., Sadowska-Bartosz I. (2018). Origin and pathophysiology of protein carbonylation, nitration and chlorination in age-related brain diseases and aging. Aging (Albany NY).

[65] Fu L., Chen Y., Xu C., Wu T., Guo H., Lin Z., Wang R., Shu M. (2020). 3D-QSAR, HQSAR, molecular docking, and new compound design study of 1, 3, 6-trisubstituted 1, 4-diazepan-7-ones as human KLK7 inhibitors. Med. Chem. Res..

[66] Shah B.M., Modi P., Trivedi P. (2021). Pharmacophore-based virtual screening, 3D-QSAR, molecular docking approach for identification of potential dipeptidyl peptidase IV inhibitors. J. Biomol. Struct. Dyn..

