# Supplementary material for: Molecular Design and Mechanism Analysis of Phthalic Acid Ester Substitutes: Improved Biodegradability in Processes of Sewage Treatment and Soil Remediation
Source: Toxics. 2022 Dec 13;10(12):783. doi: 10.3390/toxics10120783 (PMC9781866; doi:10.3390/toxics10120783)

toxics

Molecular design and Mechanism Analysis of Phthalic Acid Ester Substitutes: Improved Biodegradability in Processes of Sewage Treatment And Soil Remediation

Shuhai Sun[1,†], Qilin Zuo[1,†], Meijin Du[2,†], Yu Li[2,*]

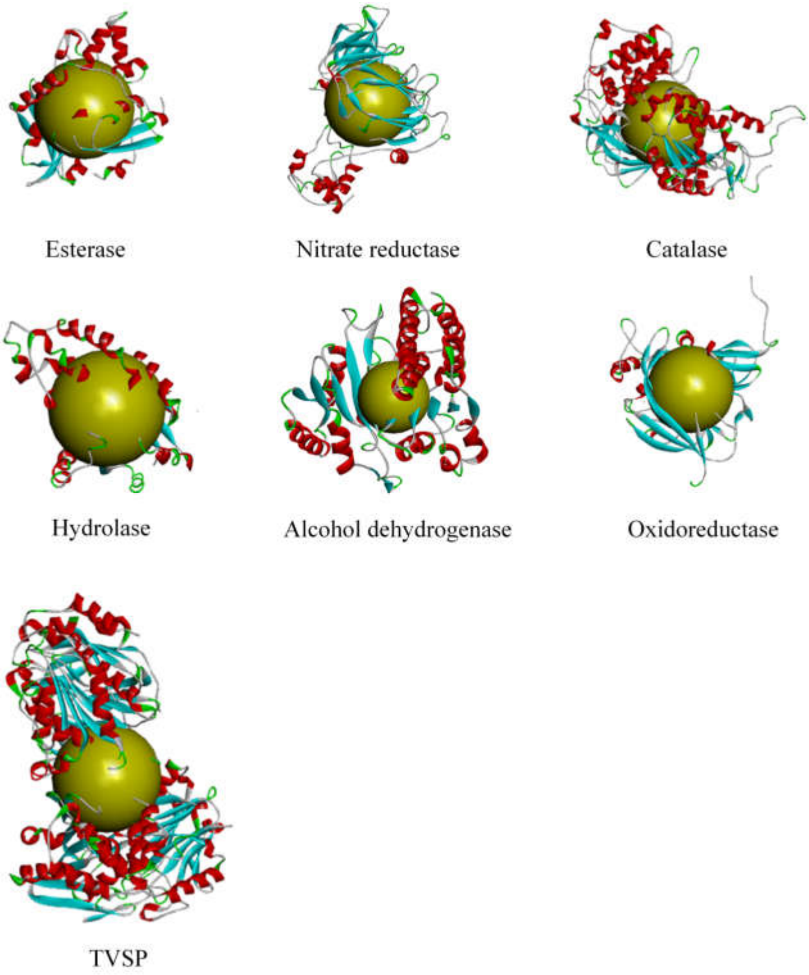

Figure S1. The docking site of proteins.

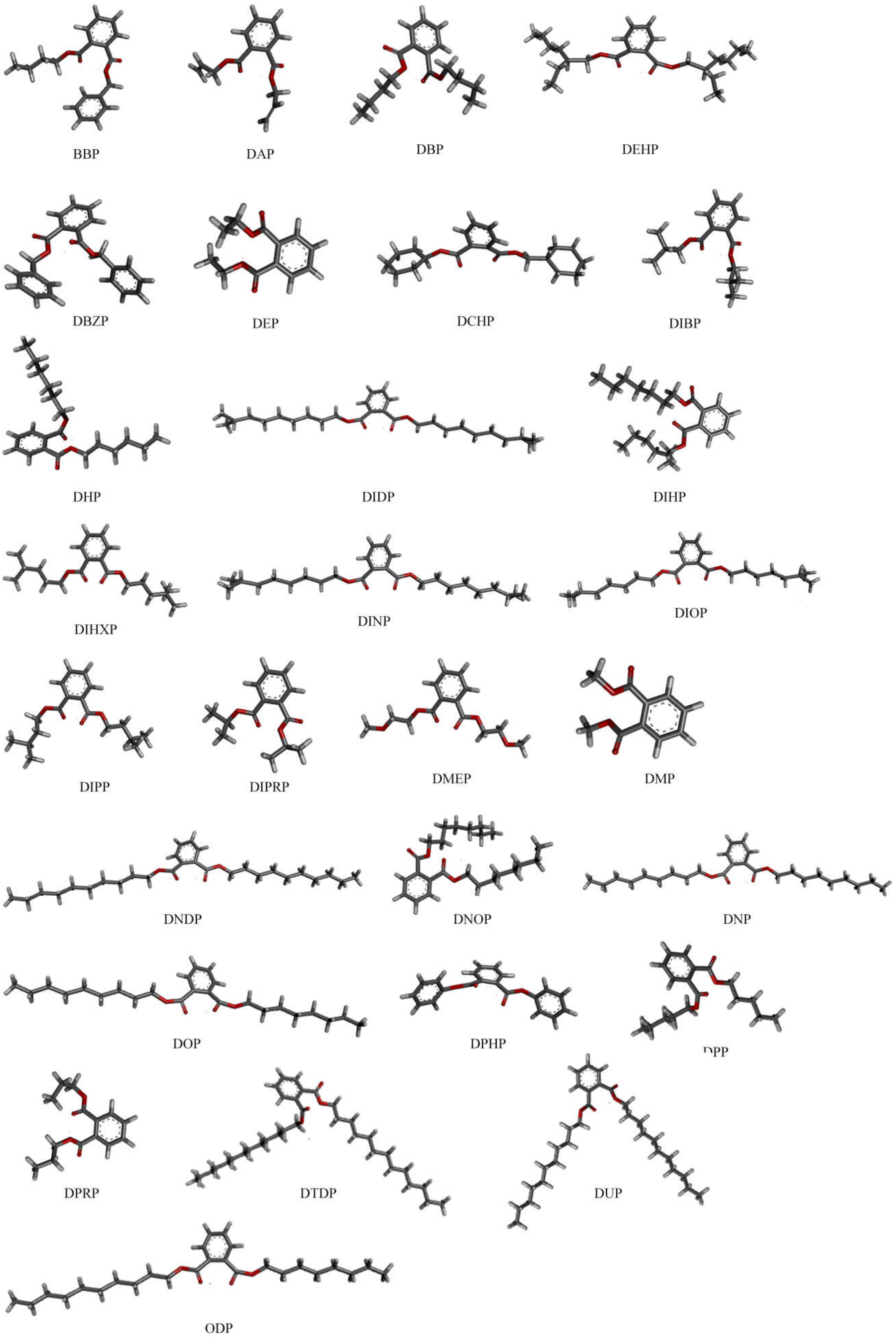

Figure S2. Molecular structure diagram of PAEs.

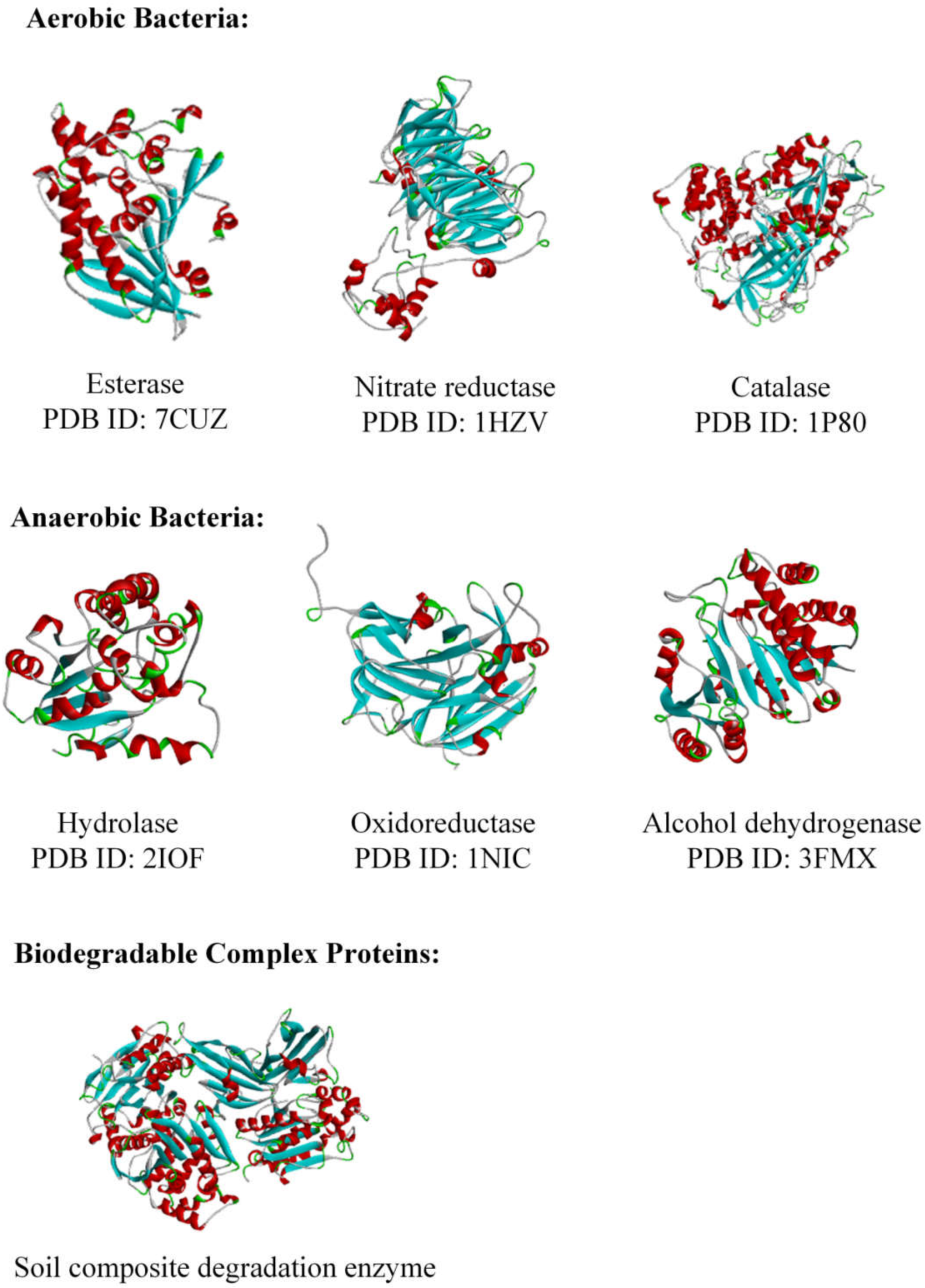

Figure S3. The structure of proteins.

Table S1. Molecular structure of DBP and DEHP substitutes.

Molecular	Chemical structure diagram	Molecular	Chemical structure diagram
DBP-1	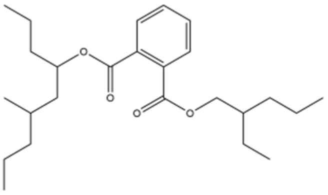	DEHP-1	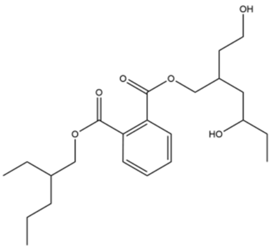

DBP-2
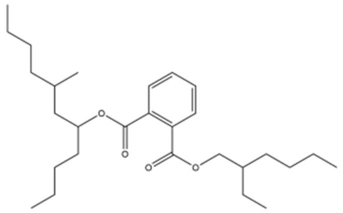

DEHP-2
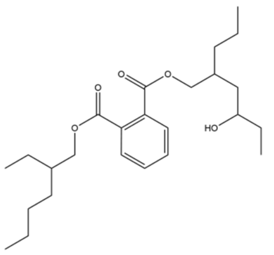

DBP-3
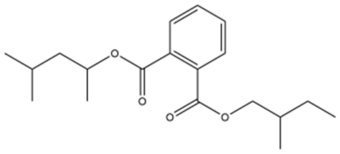

DEHP-3
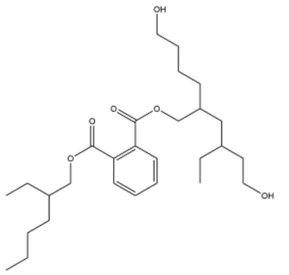

DBP-4
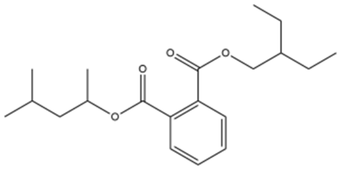

DEHP-4
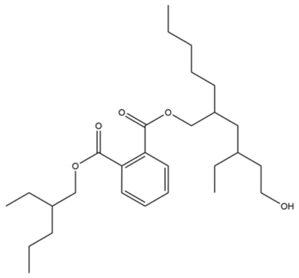

DBP-5
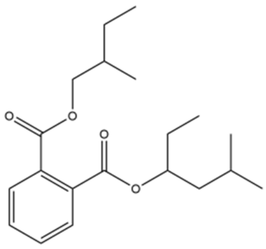

DEHP-5
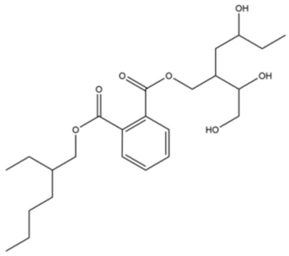

DBP-6
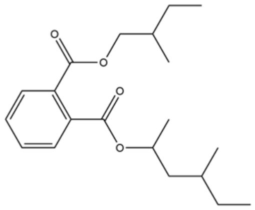

DEHP-6
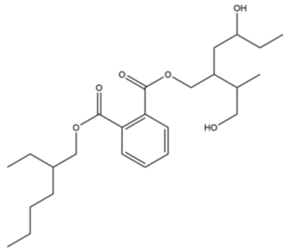

DBP-7
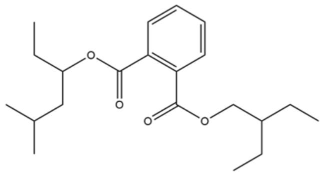

DEHP-7
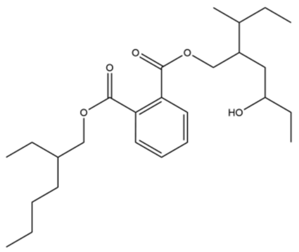

DBP-8

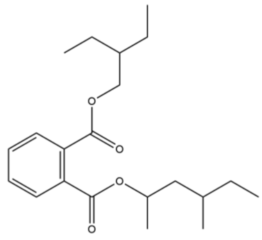

DEHP-8

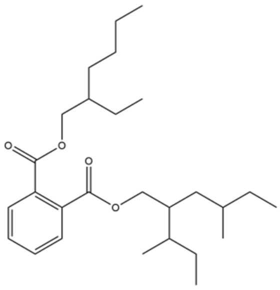

DBP-9

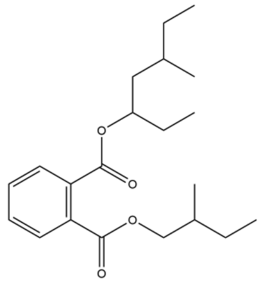

DEHP-9

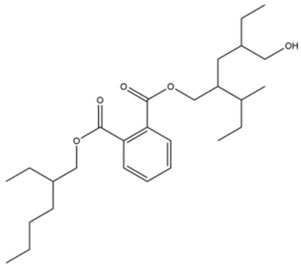

DBP-10

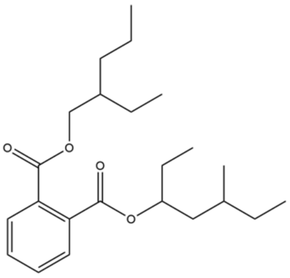

DEHP-10

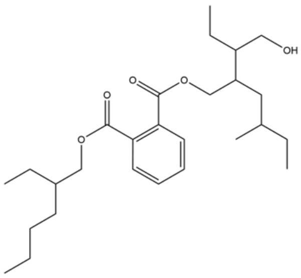

DBP-11

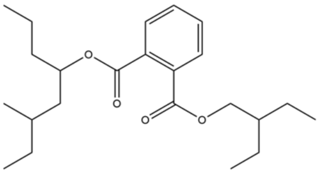

DEHP-11

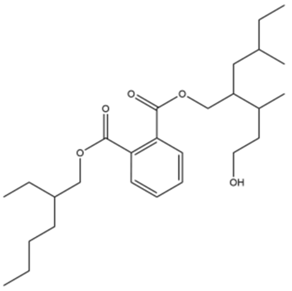

DBP-12

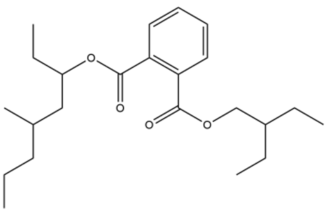

DEHP-12

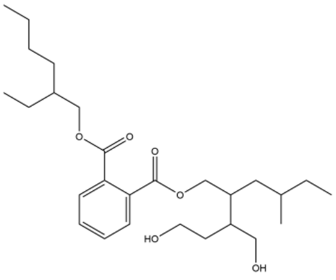

DBP-13

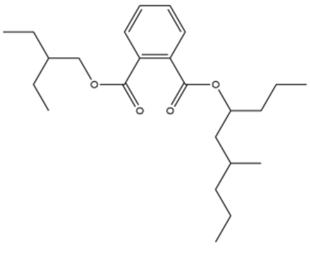

DEHP-13

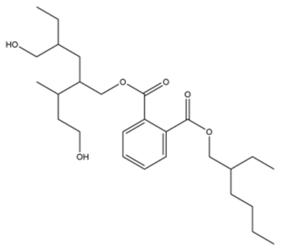

DBP-14

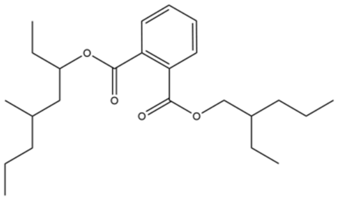

DEHP-14

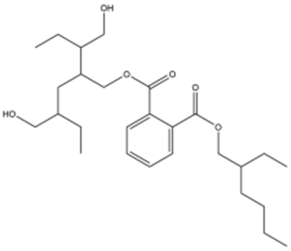

DBP-15

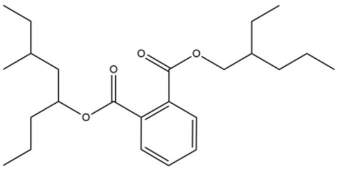

DEHP-15

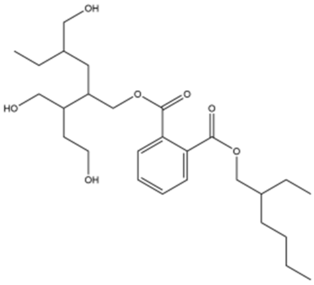

DBP-16

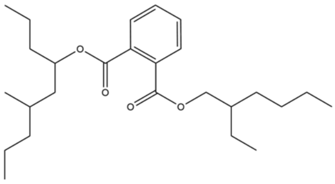

DEHP-16

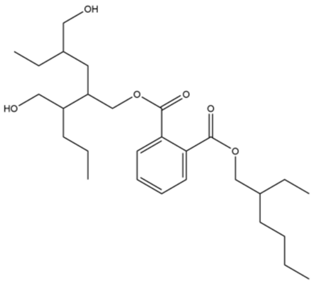

DBP-17

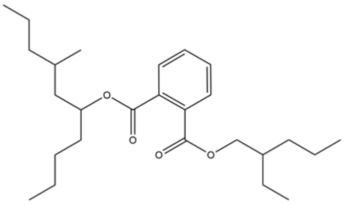

DEHP-17

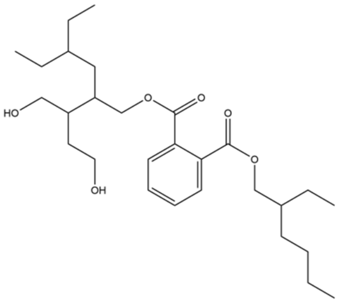

DBP-18

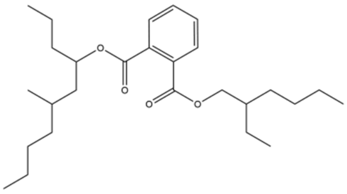

DEHP-18

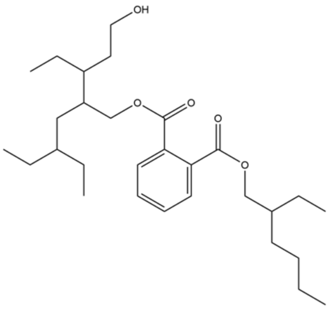

DBP-19

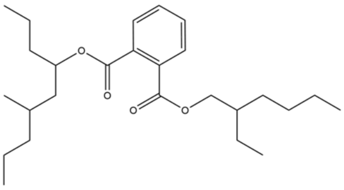

DEHP-19

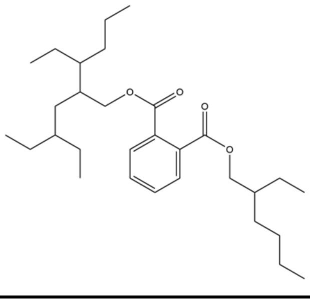

Supplement: Supplementary file 1 [file toxics-10-00783-s001.zip › toxics-2078637-supplementary.pdf]
